# Supplementary material for: Novel Signposts on the Road from Natural Sources to Pharmaceutical Applications: A Combinative Approach between LC-DAD-MS and Offline LC-NMR for the Biochemical Characterization of Two Hypericum Species (H. montbretii and H. origanifolium)
Source: Plants (Basel). 2023 Feb 1;12(3):648. doi: 10.3390/plants12030648 (PMC9921756; doi:10.3390/plants12030648)
Supplement: Supplementary file 1 [file plants-12-00648-s001.zip › plants-2171646-supplementary.pdf]

Ipericum 2023 NMR

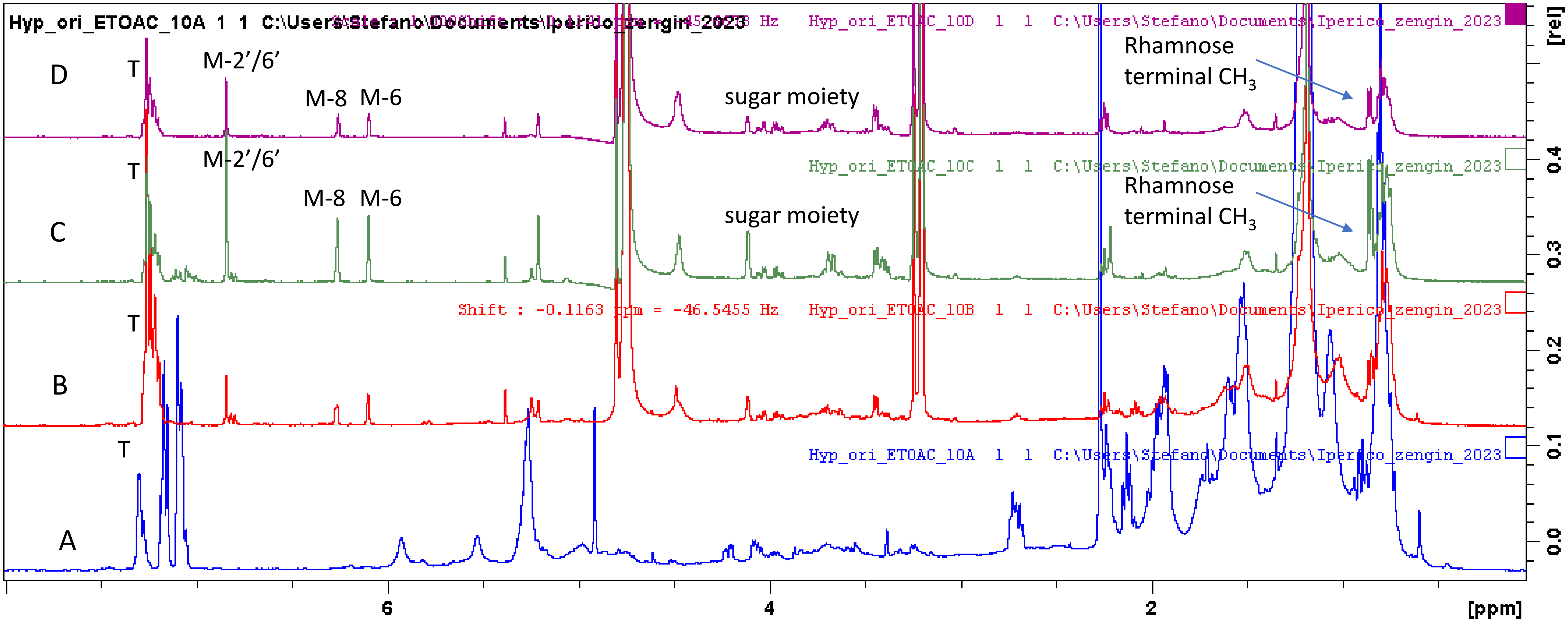

Figure S1: superimposition of the Ethyl Acetate fraction of *H. origanifolium.*

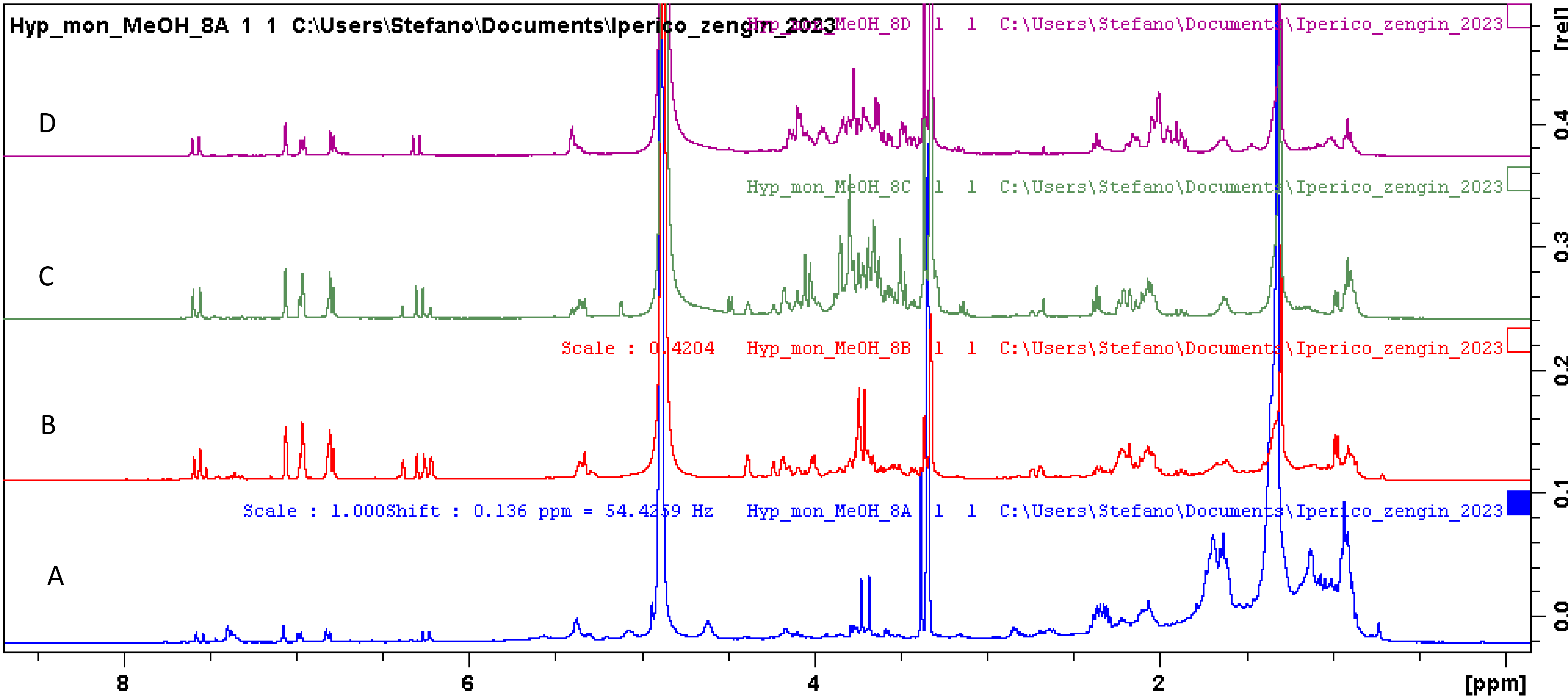

Figure S2: superimposition of the methanol fraction of *H. montbretii.*

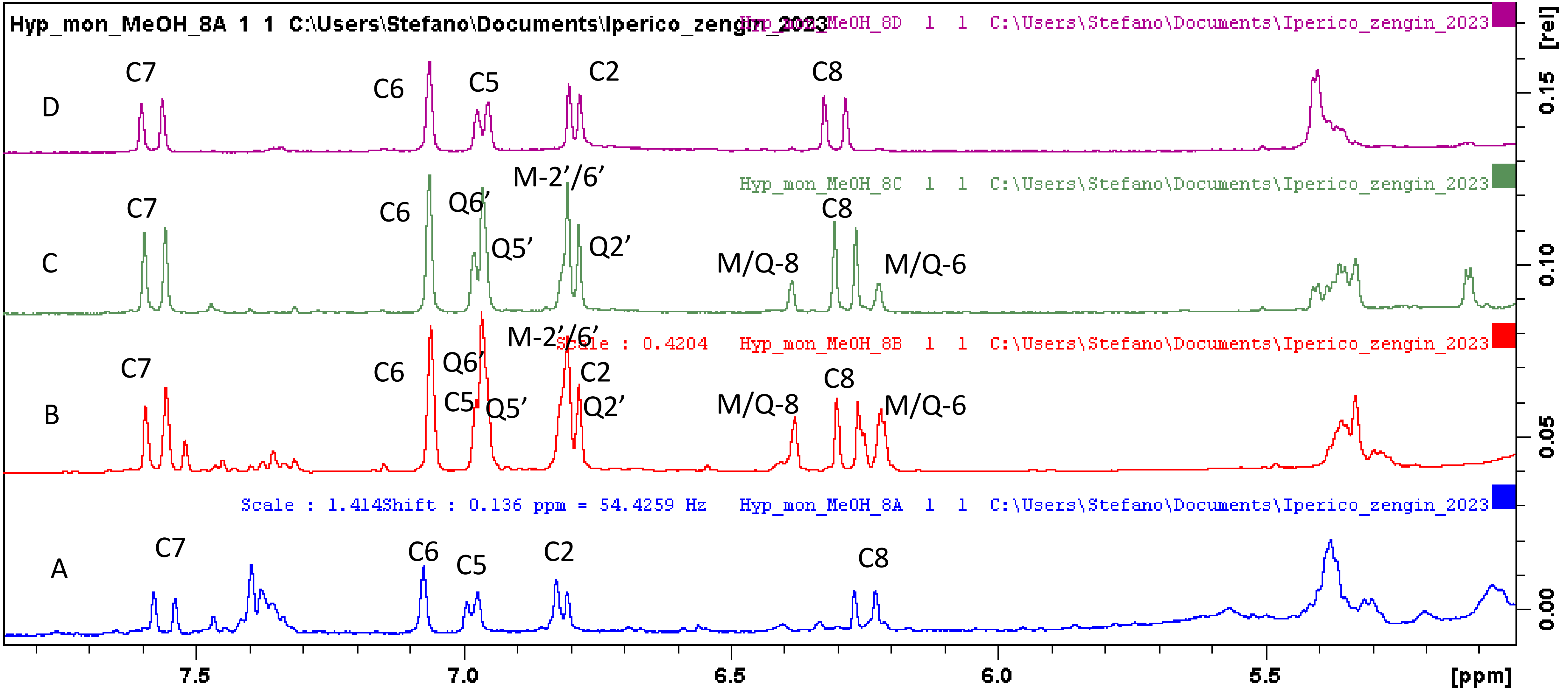

Figure S3: superimposition of the methanol fraction of *H. montbretii* enlargment of spectral region 4,5-8,0 ppm.

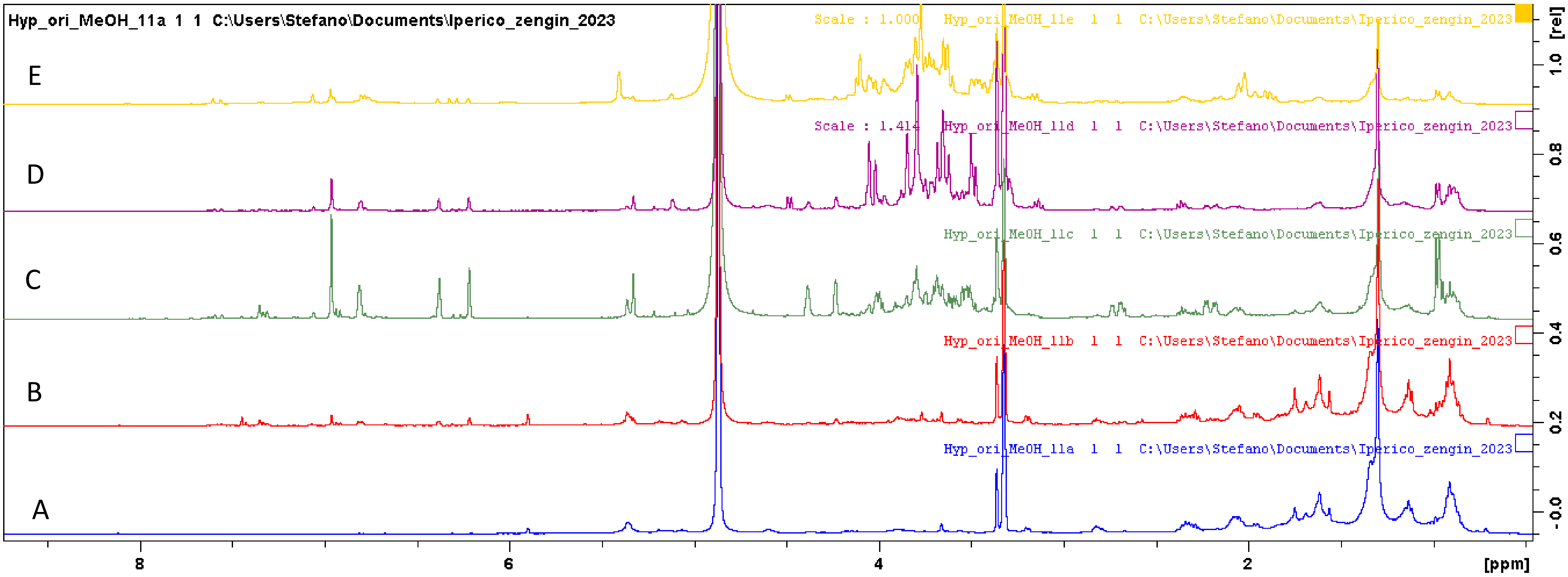

Figure S4: superimposition of the methanol fraction of *H. origanifolium.*

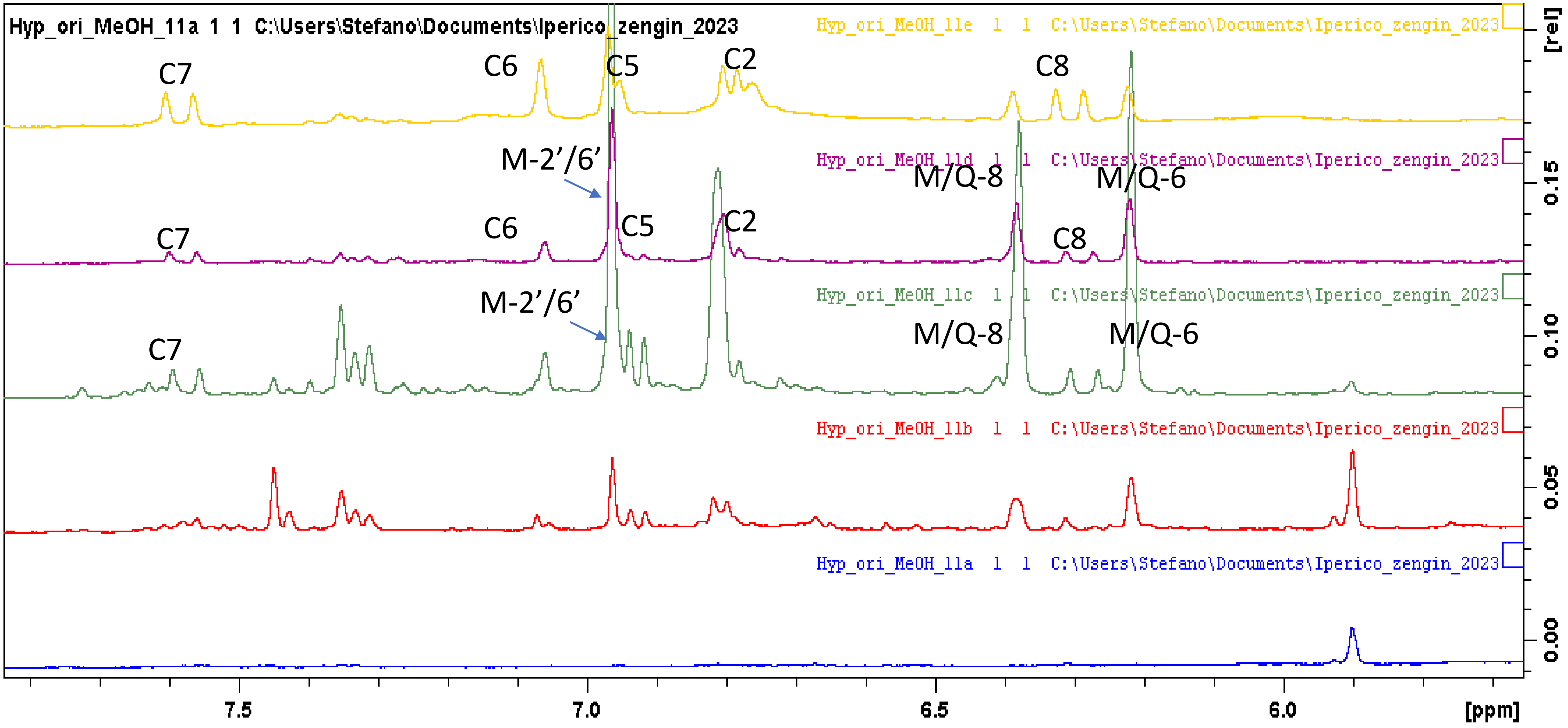

Figure S5: superimposition of the methanol fraction of *H. origanifolium* enlargment of spectral region 4,5-8,0 ppm.

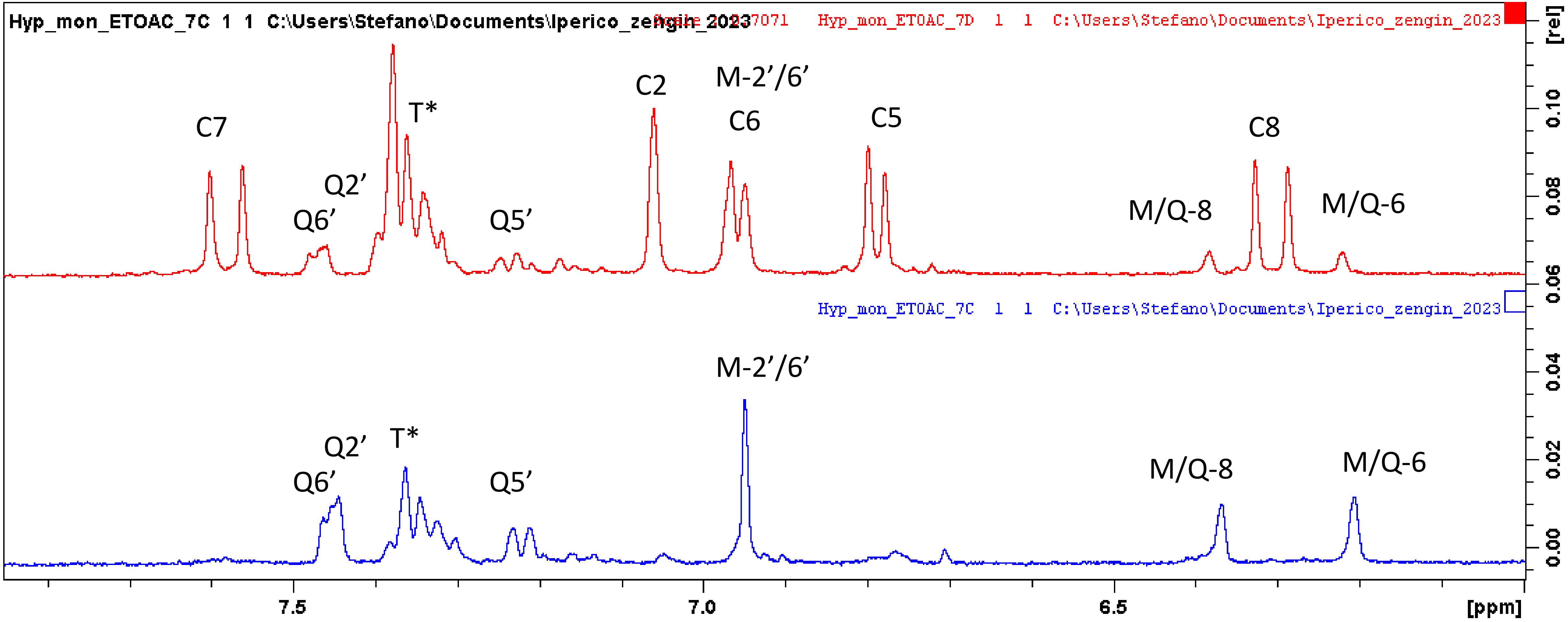

Figure S6: superimposition of the ethyl acetate fraction of *H. origanifolium* enlargment of spectral region 4,5-8,0 ppm.

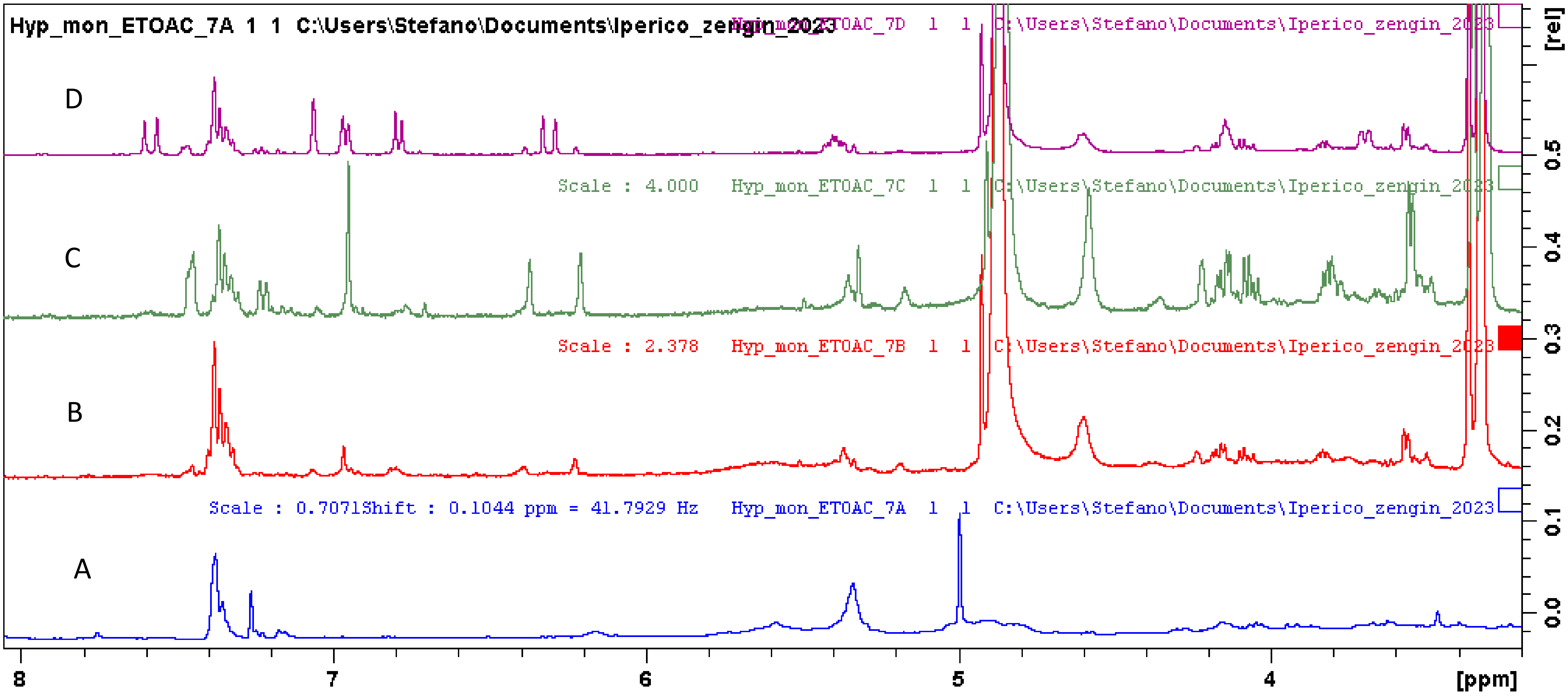

Figure S7: ethyl acetate fractions of *H. montbretii.*

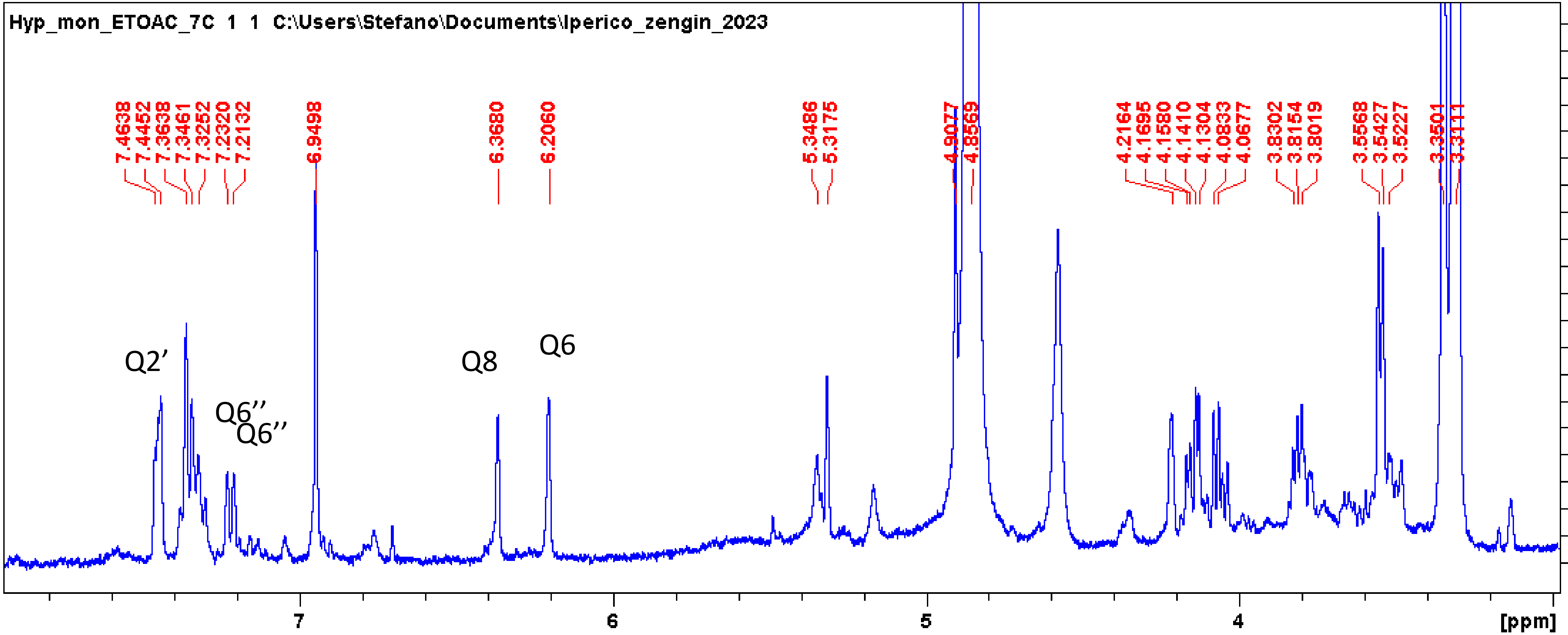

Figure S8: ethyl acetate fraction C of *H. montbretii.*

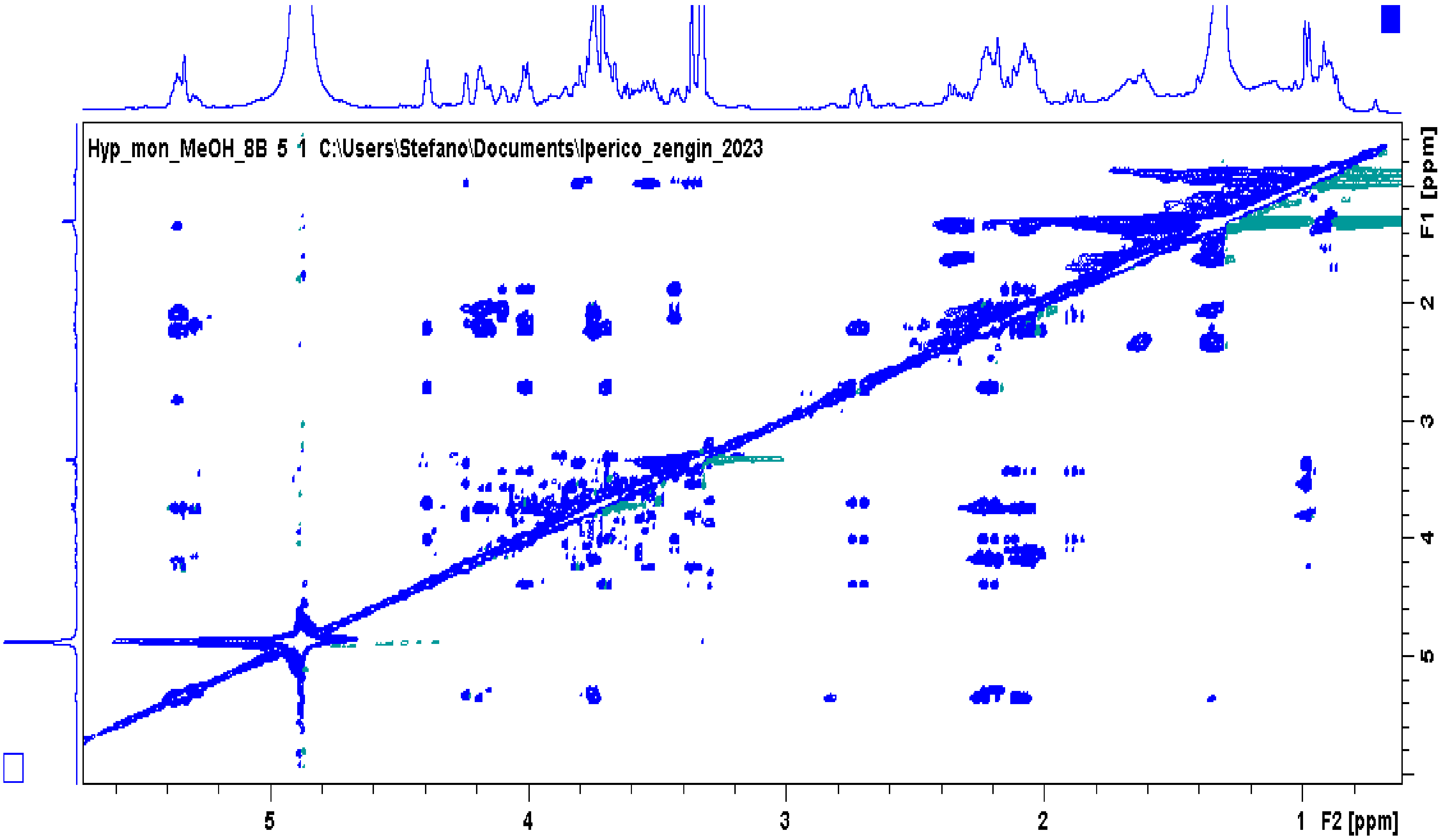

Figure S9: COSY spectrum of methanol fraction B of *H. montbretii.*

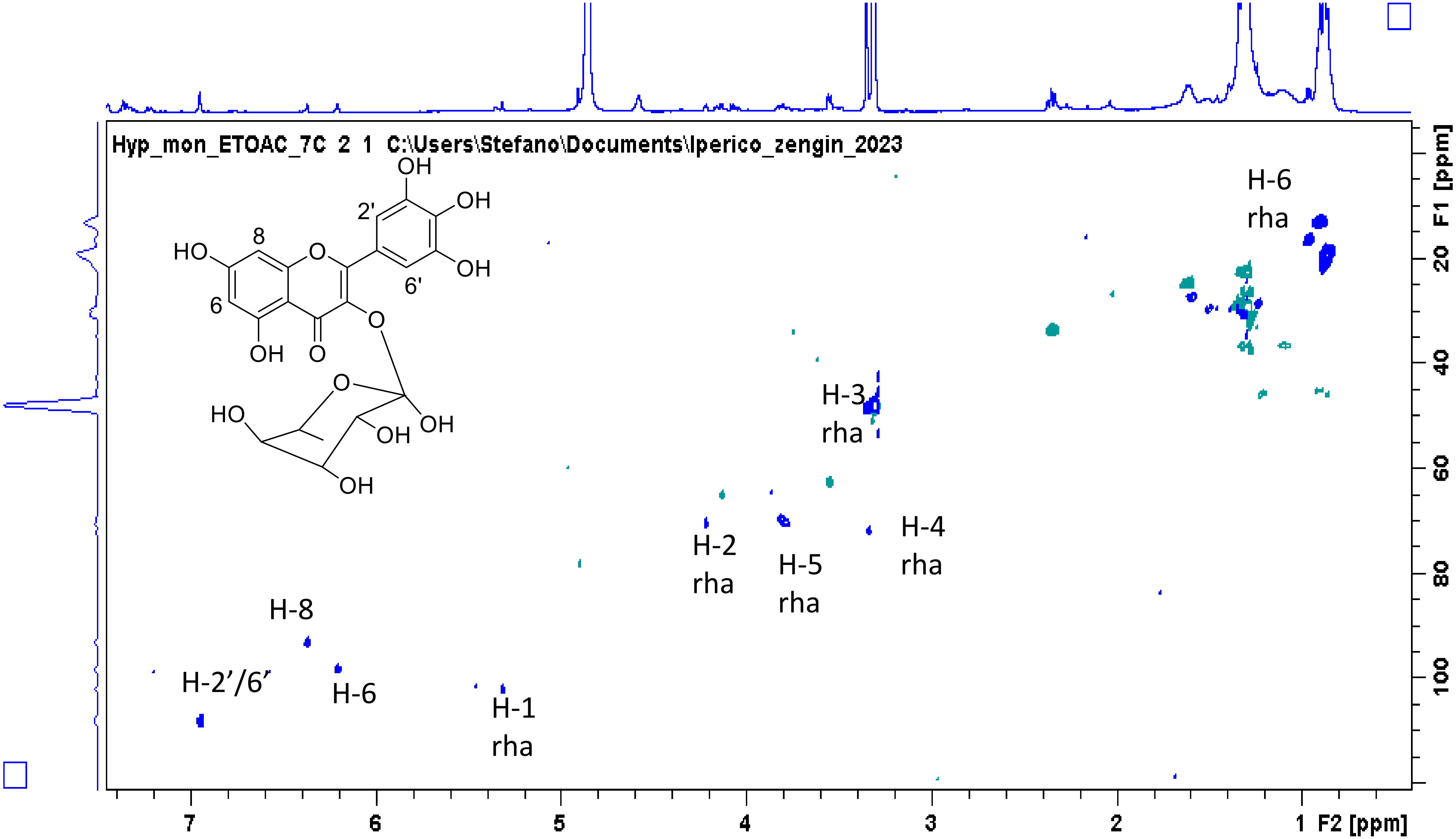

Figure S10: HSQC of fraction C of ethyl acetate extract of *H. montbretii* assignments of myricetin-3-O-rhamnoside.

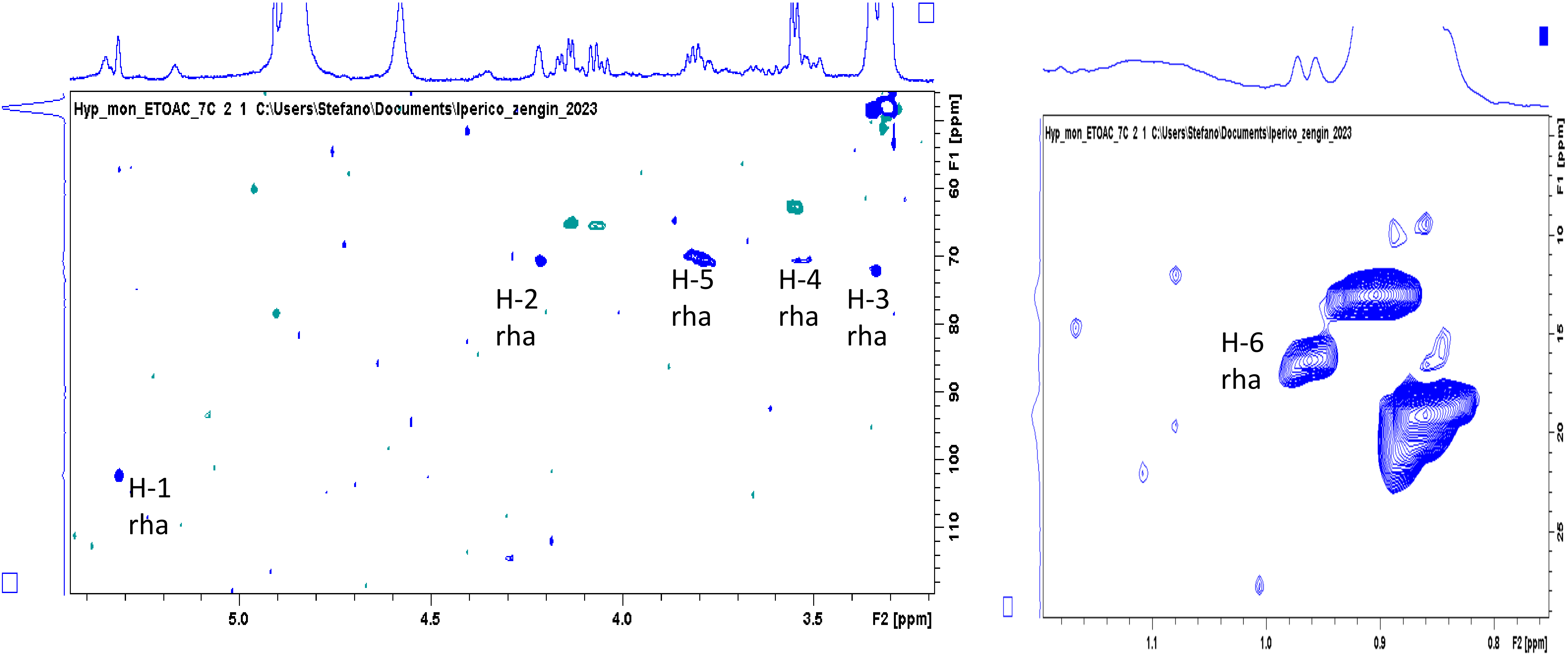

Figure S11: HSQC of fraction C of ethyl acetate extract of *H. montbretii* assignments Detail of NMR assignments of rhamnopyranosil unit of myricetin.

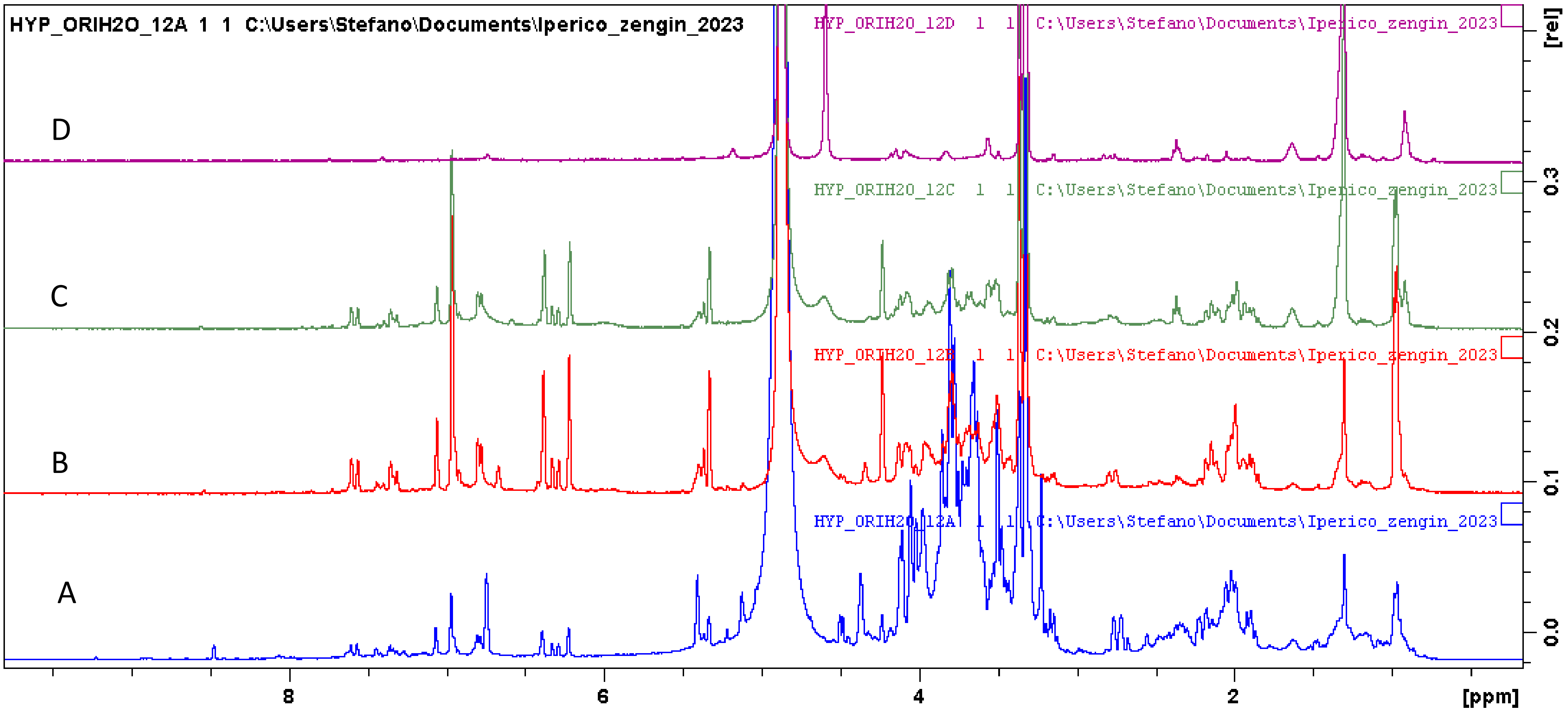

Figure S12: water extract fractions of *H. origanifolium.*

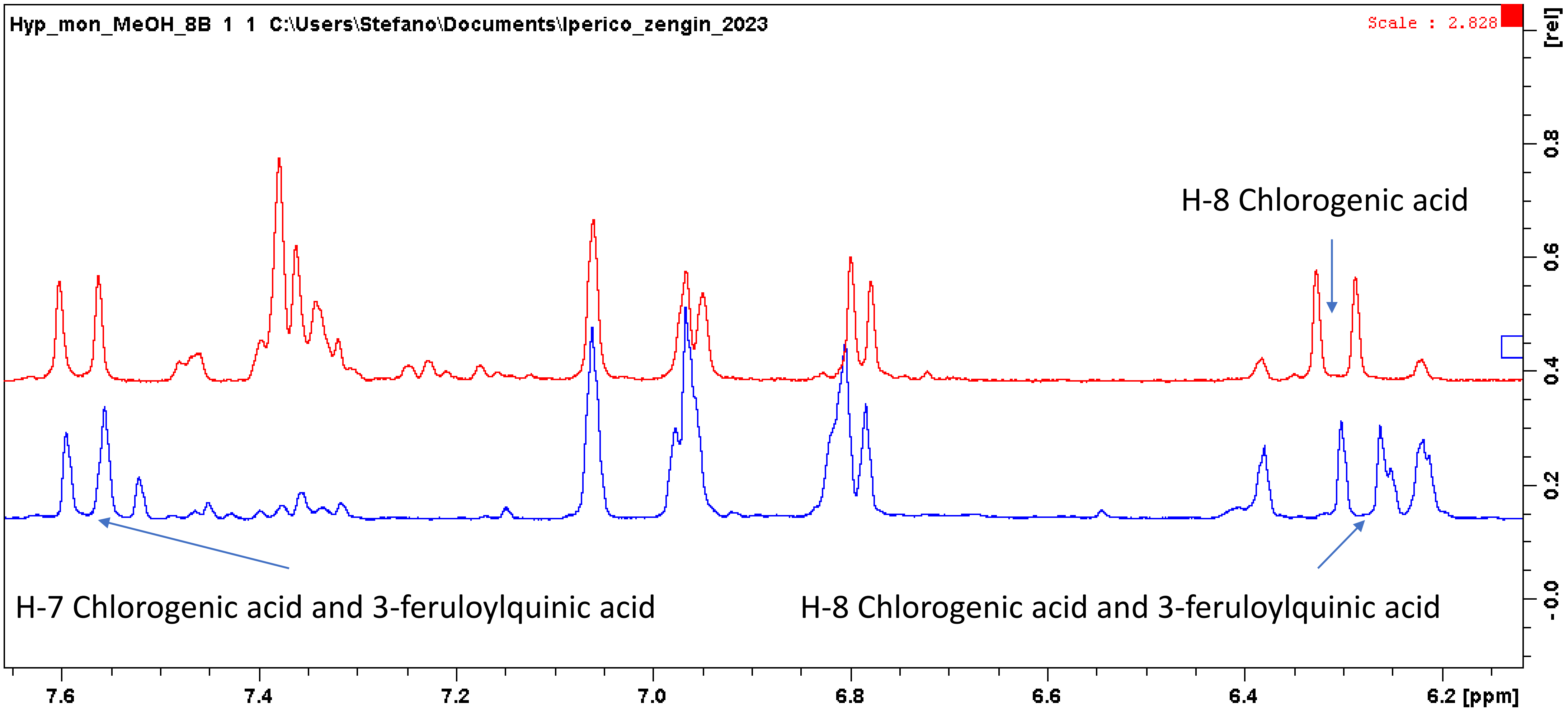

Figure S13: Detail of H-NMR related to methanol fraction B of H. montbretii indicated different hydroxycinnamic acid derivatives.

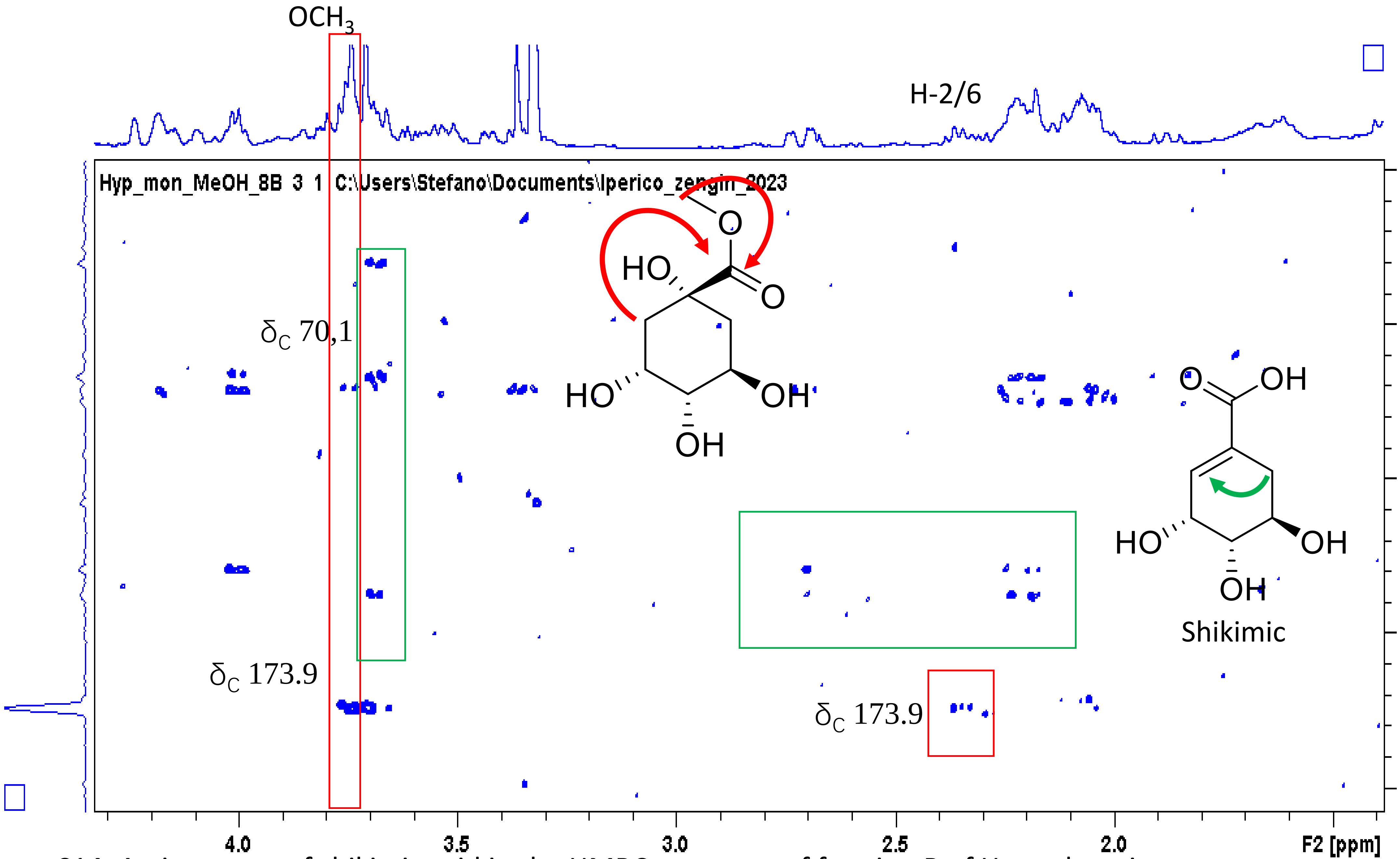

Figure S14: Assignments of shikimic acid in the HMBC spectrum of fraction B of H monbretti.

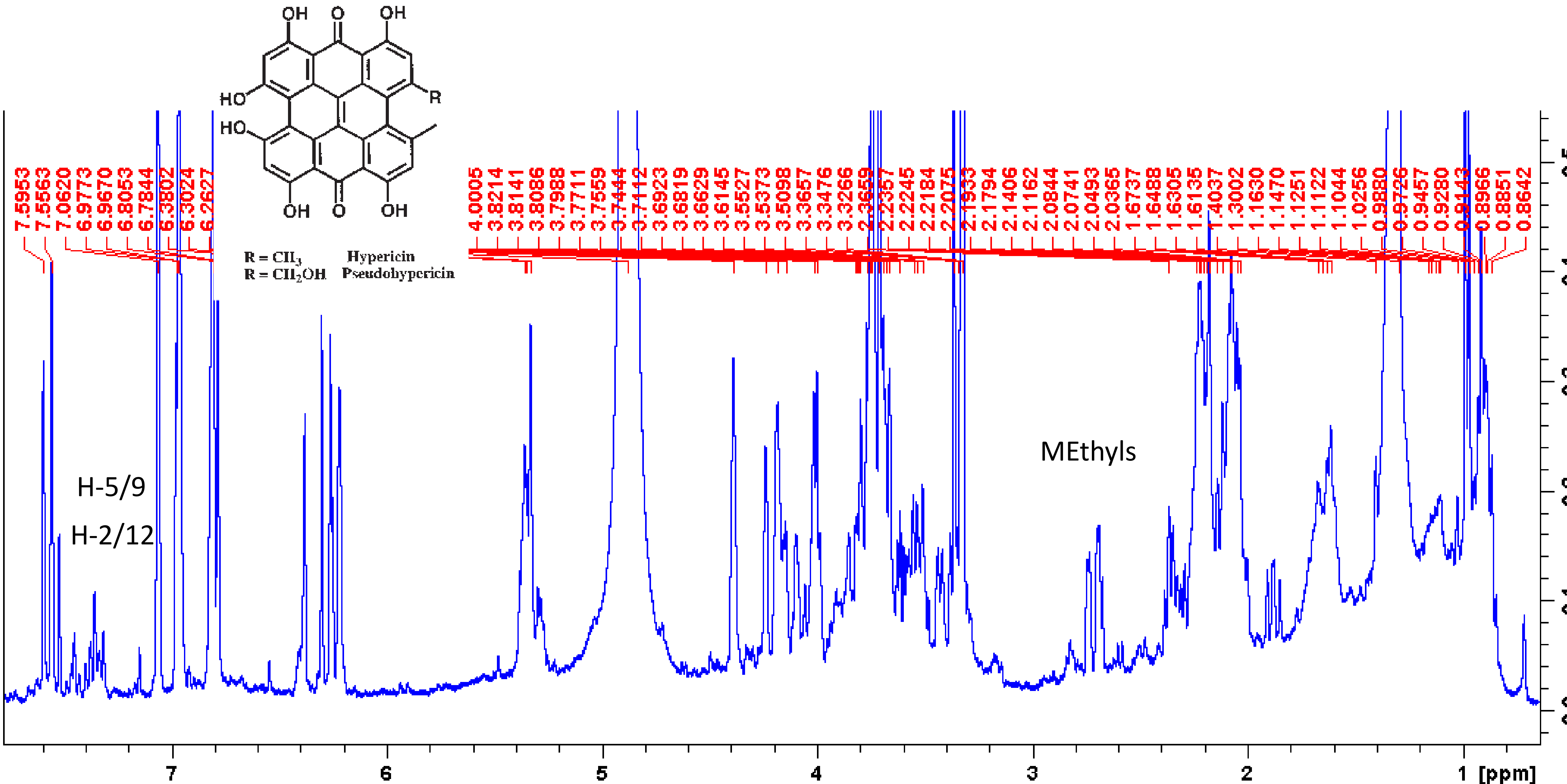

Figure S15: Assignments of proton signals ascribable to anthraquinone derivatives.

Antraquinone derivatives diagnosic aromatic signals

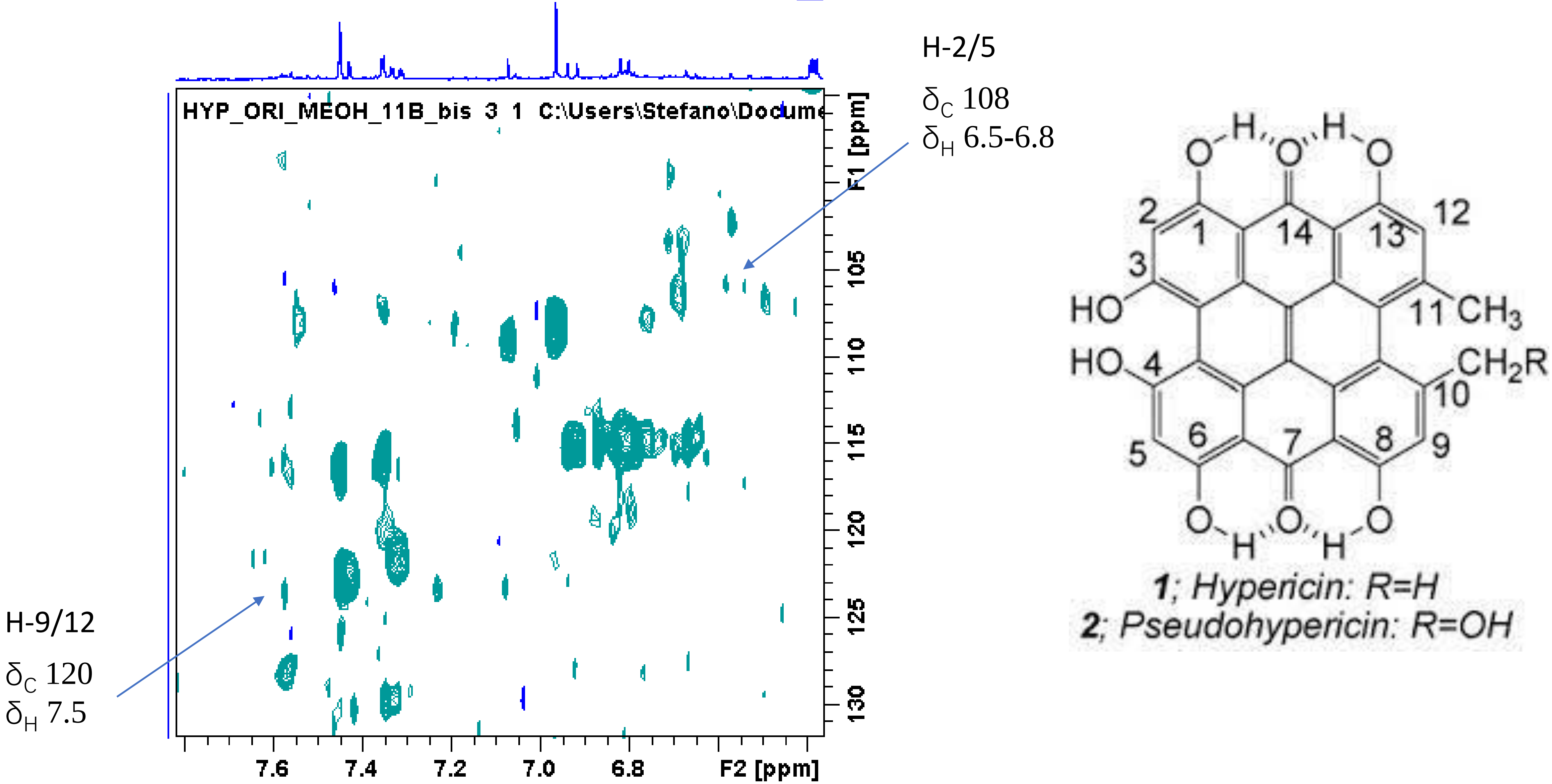

Figure S16: HSQC of H origanifolium fraction B with indicated the antracene assignments.
